# Variability in the Composition and Antioxidant Status of Milk of Polish Women Breastfeeding up to 2 Years

**DOI:** 10.3390/nu18020314

**Published:** 2026-01-19

**Authors:** Agnieszka Chrustek, Elena Sinkiewicz-Darol, Katarzyna Łubiech, Dorota Olszewska-Słonina, Agnieszka Dombrowska-Pali

**Affiliations:** 1 Department of Pathobiochemistry and Clinical Chemistry, Faculty of Pharmacy, L. Rydygier Collegium Medicum in Bydgoszcz, Nicolaus Copernicus University in Toruń, M. Curie-Skłodowska 9 St., 85-094 Bydgoszcz, Poland; 2 Human Milk Bank, Ludwik Rydygier Provincial Polyclinical Hospital in Toruń, św. Józefa 53-59 St., 87-100 Torun, Poland; 3 Department of Physiology and Toxicology, Faculty of Biological Sciences, Kazimierz Wielki University, Chodkiewicza 30 St., 85-064 Bydgoszcz, Poland; 4 Department of Perinatology, Gynecology and Gynecological Oncology, Faculty of Health Sciences, L. Rydygier Collegium Medicum in Bydgoszcz, Nicolaus Copernicus University in Toruń, Łukasiewicza 1 St., 85-821 Bydgoszcz, Poland

**Keywords:** human milk, antioxidant status, hormones, infants, lactation

## Abstract

Background/Objectives: Breastfeeding has accompanied women since the beginning of time and, according to anthropological research, naturally ends between the age of 2 and 6. WHO (World Health Organization) recommends exclusive breastfeeding for about the first 6 months, with continued breastfeeding along with introducing appropriate complementary foods for up to 2 years of age or longer. Despite the increasing promotion of breastfeeding, women do not comply with the WHO guidelines and give up exclusive breastfeeding quickly, and long-term breastfeeding mothers still struggle with a lack of understanding in society. Methods: This work aims to expand the knowledge on the composition and antioxidant status of the milk of mothers breastfeeding past 12 months. Results: The basic composition of human milk changes depending on the stage of lactation. In women breastfeeding for more than a year, an increase in fat (*p* < 0.001) and magnesium (*p* < 0.001) was observed. A decreased concentration of leptin (*p* = 0.001), iron (*p* < 0.001), and iron-reducing capacity (*p* < 0.001) was also observed compared to milk from the initial stage of lactation. Conclusions: The milk of women breastfeeding for more than 12 months is a valuable food for the baby, maintaining its protection against free radicals and providing adequate nutrients.

## 1. Introduction

Breastfeeding has accompanied the human species from the very beginning. Bone collagen studies conducted using the methods of carbon and nitrogen isotope analysis and analysis of the skeletal remains of children and adults have shown that in the prehistoric period, mothers breastfed until the child was 3–4 or even 6 years old [1,2,3].

Human milk has a rich nutritional composition that changes depending on maternal, geographical, and perinatal factors and lactation phases. Increasingly, human milk is considered a medicine due to its wide range of properties, including anti-inflammatory, anti-infective, immunomodulatory, and antioxidant [4,5,6,7,8,9,10,11,12,13,14,15].

WHO (World Health Organization) recommends exclusive breastfeeding for about the first 6 months, with continued breastfeeding along with introducing appropriate complementary foods for up to 2 years of age or longer [16]. Breastfeeding has beneficial effects for both the baby and the mother. It has been shown to protect the mother against cancers such as endometrial and ovarian cancer [17,18] and to protect the child against future metabolic diseases as well as strengthening the immune system [19,20].

Currently, the breastfeeding initiation rate is increasing but remains low. The main reasons for discontinuing breastfeeding include nipple inflammation and injury and low milk supply. Additional contributing factors are the mother’s living conditions, public health policies, insufficient support for breastfeeding women, inadequate training of medical staff, and the availability of formula milk [21,22]. Breastfeeding rates are also influenced by breastfeeding education for pregnant women. Prenatal education has been identified as an integral component of WHO’s 10 Steps to Supportive Breastfeeding [23]. It increases the knowledge of both women and their partners about breastfeeding. Family members who are well-informed about breastfeeding are more likely to offer support to breastfeeding mothers, which in turn increases their confidence in breastfeeding [24]. Mothers who are knowledgeable about breastfeeding and have a positive attitude towards breastfeeding are more likely to initiate breastfeeding and continue it for a longer period. The results indicate a general correlation between prenatal breastfeeding education and increased rates of postpartum breastfeeding initiation [25].

According to UNICEF, in developed countries more than one in five children is not breastfed, while in low- and middle-income countries almost all children are breastfed. Globally, the exclusive breastfeeding rate among children aged 0–5 months between 2017 and 2023 was 46% [26]. In 2022 in the United States, 85.7% of women initiated breastfeeding, more than half (62.1%) were still breastfeeding at 6 months, and more than one third (40.8%) continued at 12 months [27].

In Poland, according to the data of the Central Statistical Office (2014), 98% of women initiated breastfeeding after childbirth, while in the sixth week only 46% of mothers were breastfeeding. From 2 to 6 months of age, 42% of women breastfed their children; only 17% continued at 9 months, and 11.9% were still providing human milk at 12 months [28].

Mothers that continue BF (breastfeeding) past 6 months often face negative attitudes and criticism, stating that breastfeeding past a certain time is pointless and does not have any nutritional value [29]. It is believed that breastfed children are at risk of caries, malocclusion [30], and mental, sexual, and emotional problems. Of course, none of the above-mentioned allegations are supported by scientific research. Unfortunately, these beliefs prevail over the knowledge of doctors, pediatricians, and dentists as well. This work aims to expand the knowledge on the composition and high antioxidant status of the milk of mothers that breastfeed past 12 months.

The composition of human milk produced over a period of 6 months is widely researched and described, while there is little information on the composition of human milk produced after one year. The aim of this study was to analyze the basic components of human milk depending on the stage of lactation. Specific objectives included assessing the antioxidant status, selected hormones, and micro- and macronutrients in the milk of breastfeeding women, taking into account four breastfeeding time periods (2–7 weeks, 8–24 weeks, 25–48 weeks, and 49–96 weeks).

The innovative nature of this work stems from the fact that, for the first time in Poland, the composition and antioxidant status of milk from women living in the same region were analyzed, taking into account a wide lactation period. Furthermore, there are no studies analyzing the composition and antioxidant status of milk from women with such a long lactation period (up to 2 years).

## 2. Materials and Methods

The scientific research was approved by the Bioethics Committee of the Nicolaus Copernicus University in Toruń, Collegium Medicum in Bydgoszcz, nos. KB437/2018 (22 May 2018) and KB348/2022 (21 June 2022).

### 2.1. Study Groups

**Figure 1:**
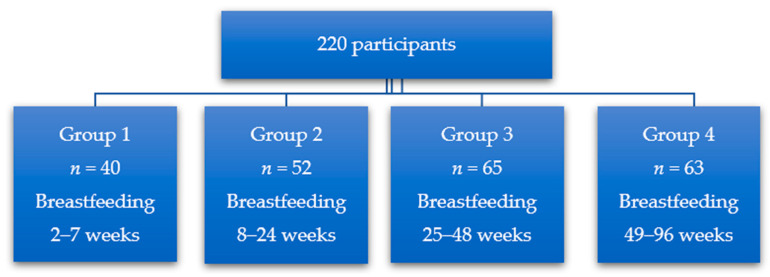
Division of study participants into study groups according to lactation period.

A total of 220 women (Figure 1) from the Kujawsko-Pomorskie Voivodeship (Poland) were recruited for the study. The women gave birth at 37–40 weeks (HBD (hebdomas)), were non-smokers, and took no medications, antibiotics, or probiotics. The women were healthy, with low physical activity, supplementing vitamin D (2000 IU), omega 3 acids (DHA (docosahexaenoic-acid)—800 mg; EPA (eicosapentaenoic acid)—68 mg) and folic acid (pteroylmonoglutamic acid—200 μg; L-calcium methylfolate—200 μg). The women did not follow diets and had no dietary restrictions or any food intolerances.

All study participants gave written consent to participate in the research project. During the recruitment process, all participants completed a questionnaire on maternal factors affecting the human milk composition. The survey included data such as age, height, weight, BMI, mother’s health status (occurrence of diseases during pregnancy and breastfeeding period), mother’s diet (e.g., veganism, vegetarianism), lifestyle (physical activity, medications, supplements, stimulants), and information regarding childbirth.

### 2.2. Materials for the Study

Human milk samples were collected at the Department of Pathobiochemistry and Clinical Chemistry of the Collegium Medicum in Bydgoszcz, Poland.

Pooled human milk samples were collected during the day (40 mL). Participants pumped milk at home using an electric breast pump. Mothers pumped 5 mL of milk before feeding the baby, then fed the baby and pumped 5 mL of milk again after feeding at agreed intervals.

Each woman expressed milk using a breast pump according to the daily milk collection protocol four times a day, from four time intervals: 06:00–12:00, 12:00–18:00, 18:00–24:00, and 24:00–6:00. Each portion of milk was poured into one collective bottle.

The collected samples were mixed and stored in the fridge. The material was delivered to university laboratories by participants within 24 h of collection, then it was portioned into 2 mL Eppendorf tubes (MedLab, Raszyn, Poland) and frozen at −20 °C and then −80 °C for further analysis. The material was stored in such conditions for less than 6 months.

### 2.3. Determination of the Basic Composition of Human Milk

The basic composition of human milk includes fat [g/100 mL], total protein [g/100 mL], crude protein [g/100 mL], carbohydrates [g/100 mL], total solids [g/100 mL], and energy value [kcal /100 mL]. The determination was performed on human milk samples using the MIRIS HMA analyzer (MIRIS AB, Uppsala, Sweden) according to the manufacturer’s procedure. Prior to analysis, human milk samples were heated at 40 °C in a thermostatic bath (BIONOVO, Legnica, Poland) and then homogenized using the MIRIS Sonicator [1.5 s/mL]. Each sample was analyzed in triplicate.

### 2.4. Determination of the Concentration of Hormones in Human Milk

In order to determine the level of adiponectin, leptin, and cortisol in human milk, commercial enzyme immunoassays were used according to the manufacturer’s procedure [Human Leptin ELISA (BioVender, Brno, Czech Republic), Human Adiponectin/Acrp30 (DuoSet ELISA, R&D systems, Minneapolis, MN, USA), Cortisol Elisa (DiaMetra., Spello, Italy)]. Breast milk samples were centrifuged for 10 min at 10,000 rpm, and then 100 µL of supernatant was added to the plate. Tests were conducted in accordance with the manufacturer’s procedure. Each sample was analyzed in triplicate.

### 2.5. Chemicals

Chemicals: DPPH• (2,2-diphenyl-1-picrylhydrazyl, Sigma Aldrich, Saint Louis, MO, USA); methanol (POCH, Gliwice, Poland); Trolox (Sigma Aldrich, Saint Louis, MO, USA); acetate buffer (pol-aura, Dywity, Poland); TPTZ (2,4,6-Tris(2-pyridyl)-s-triazine, Sigma Aldrich, Saint Louis, MO, USA); FeCl_3_*6H_2_O (POCH, Gliwice, Poland); ascorbic acid (Chempur, Piekary Śląskie, Poland); Carrez I (POCH, Gliwice, Poland); Carrez II (POCH, Gliwice, Poland); Folin–Ciocalteu’s reagent (POCH, Gliwice, Poland); sodium carbonate (POCH, Gliwice, Poland); gallic acid (Merck, Darmstadt, Germany); acetonitrile (POCH, Gliwice, Poland).

### 2.6. Determination of the Antioxidant Activity of Human Milk Using the DPPH• Radical (2,2-Diphenyl-1-Picrylhydrazyl)

In order to determine the level of DPPH, the method of Atanassova et al. (2011) was used with minor modifications [31].

Before the experiment, a 100 µM solution of DPPH• (2,2-diphenyl-1-picrylhydrazyl, Sigma Aldrich, Saint Louis, MO, USA) was prepared by dissolving 4 mg of the reference substance in 100 mL of methanol (POCH, Gliwice, Poland), then 1 mL of the prepared DPPH solution was added to Eppendorf tubes and 250 µL of human milk was added, vortexed, and then incubated for 60 min in the dark at room temperature. Prior to reading, samples were centrifuged for 2 min at 1500× *g* at room temperature. Absorbance was measured at 517 nm against a methanol reference. The control sample was a 100 µM methanolic DPPH solution, which was measured at the beginning and at the end of the experiment. The solutions to obtain the curve were prepared in an analogous way, replacing 250 µL of human milk with Trolox solutions. Each sample was analyzed in triplicate.

The percentage ability of human milk to reduce the DPPH• radical was calculated:

$$\%\;inhibition=(\frac{A-Ab}{A})\times 100$$

A—control absorbance.

Ab—average absorbance value of the tested human milk.

### 2.7. Determination of the Ability of Human Milk to Reduce Fe (III) Ions

For the determination of FRAP, the method of Benzie and Strain (1999) with modifications was used [32].

Before the experiment. the following was prepared:

(a) 300 mM acetate buffer, pH 3.6;
(b) 10 mM TPTZ in 40 mM HCl;
(c) 20 mM FeCl_3_*6H_2_O.

The first step was to prepare the FRAP reagent by combining solutions a, b, and c in a ratio of 10:1:1. For the determination, a blank sample was prepared, which was the FRAP reagent, along with 100 µL of human milk samples and standards for the calibration curve. The next step was the application of 100 µL of the sample and 3 mL of FRAP reagent, vortexing, and then immediate reading of the absorbance of the sample at 0 min at 593 nm wavelength and incubation at 37 °C in a water bath for 4 min. The last step was vortexing and subsequent measurement of absorbance at the same wavelength. Each sample was analyzed in triplicate.

### 2.8. Preparation of the Calibration Curve

Six solutions of ascorbic acid (Chempur, Piekary Śląskie, Poland) with concentrations of 100–1000 µM were prepared to determine the reduction capacity of Fe (III) ions in human milk. The standard curve shows the dependence of the absorbance value of ascorbic acid on its concentration (y = 0.0006x − 0.0392; R^2^ = 0.992). FRAP concentrations are expressed as ascorbic acid equivalents (µM).

The FRAP value was calculated from the formula

$$FRAP=\frac{sample\;absorbance\;change\;from\;0\;min\;to\;4\;min}{standard\;absorbance\;change\;from\;0\;\min\;to\;4\;min}\times standard\;concentration\times 2\;[µM]$$

### 2.9. Determination of the Content of the Total Sum of Polyphenols

The method of Vazquez et al. (2015) with modifications was used for determination [33]. Milk samples were gradually thawed in the refrigerator and vortexed. The first stage of the assay was the deproteinization of samples by mixing 1 mL of human milk sample with 2 mL of 50% methanol, 100 µL of Carrez I and Carrez II reagents, and 1 mL of acetonitrile. Then the samples were vortexed and made up to 5 mL with 50% methanol. Samples were incubated at room temperature for 25 min and finally centrifuged at 4500× *g* at 4 °C for 15 min.

The total sum of polyphenols was determined in the aqueous layer of the sample by the FC method (Folin–Ciocalteu), in which 100 µL of the methanol extract was mixed with 1 mL of the previously prepared FC reagent (FC:H_2_O; 1:1. *v*/*v*) and 3 mL of 20% Na_2_CO_3_. After vortexing, the mixture was incubated for 30 min in the dark at room temperature. The absorbance at 765 nm was then measured using a UV/VIS spectrophotometer (Biosense, Wrocław, Poland). The solutions necessary to obtain the calibration curve were prepared by replacing 100 µL of methanol extract with solutions of gallic acid (Merck, Darmstadt, Germany) of known concentration. The reference sample was 100 µL of methanol. Each sample was analyzed in triplicate.

### 2.10. Calibration Curve Preparation

Six standard water solutions of gallic acid (GAE) with concentrations of 0.0–0.10 mg/mL were prepared. The total content of polyphenols was calculated on the basis of a calibration curve describing the dependence of the absorbance value on the concentration of gallic acid (y = 2.3707x + 0.1526; R^2^ = 0.995). The results are presented as gallic acid equivalents in 1000 mL human milk (mg GAE/L).

### 2.11. Determination of Vitamins and Micro- and Macroelements in Human Milk

#### 2.11.1. Iron Concentration Determination

A set of reagents from BioMaxima (Lublin, Poland) was used to determine the level of iron. Each sample was analyzed in triplicate. The determination consisted of incubation at 37 °C of the R1 reagent with a sample of centrifuged human milk, and then centrifugation (several seconds at the highest speed) and reading the absorbance at a wavelength of 590 nm against the reagent sample. The reagent sample contained distilled water instead of the test sample. Then, the reagent R^2^ was added to the supernatant and incubated for 5 min at 37 °C and the absorbance was read against the reagent test. The standard sample was made using the same principle as the test sample, with the use of a ready-made BioMaxima standard (200 µg/dL).

Iron concentration in human milk samples was calculated from the formula

$$Iron\;concentration=\frac{A2(PB)-A1(PB)}{A2(PW)-A1(PW)}\times standard\;concentration\;[µg/dL]$$

A1(PB)—absorbance of 1 test sample;

A2(PB)—absorbance of 2nd test sample;

A1(PW)—absorbance of 1 standard;

A2(PW)—absorbance of 2nd standard.

#### 2.11.2. Determination of the Concentration of Magnesium, Calcium, and Phosphorus

In order to estimate the concentration of magnesium, calcium, and phosphorus in human milk, colorimetric kits by BioMaxima (Lublin, Poland) were used. Each sample was analyzed in triplicate. The determination consisted of incubating a centrifuged human milk sample at 37 °C with a BioMaxima reagent and reading the absorbance against the reagent sample at a wavelength of 550 nm. The reagent sample contained distilled water instead of the test sample. The standard sample was made using the same principle as the test sample, with the use of a ready-made standard from BioMaxima (2 mg/dL—magnesium; 5 mg/dL—phosphorus; 10 mg/dL—calcium).

The concentration of magnesium, calcium, and phosphorus in human milk samples was calculated from the formula

$$Compound\;concentration=\frac{A(PB)}{A(PW)}\times standard\;concentration\;[mg/dL]$$

A(PB)—sample absorbance;

A(PW)—standard absorbance.

#### 2.11.3. Determination of Chloride Concentration

During the analyses, a reagent kit for direct, colorimetric determination of chlorides by Hydrex (Castello d’Argile, Italy) was used. Each sample was analyzed in triplicate. The determination consisted of incubating a centrifuged human milk sample at 37 °C with a Hydrex reagent and reading the absorbance against the reagent sample at a wavelength of 550 nm. The reagent sample contained distilled water instead of the test sample. The standard sample was made using the same principle as the test sample, with the use of a ready-made Hydrex standard (100 mmol/L).

The chloride concentration in human milk samples was calculated from the following formula:

$$Chloride\;concentration=\frac{A(PB)}{A(PW)}\times standard\;concentration\;[mmol/L]$$

A(PB)—sample absorbance;

A(PW)—standard absorbance.

### 2.12. Statistical Analysis

The Statistica 13.1 software package by StatSoft^®^ (Kraków, Poland) was used for statistical analysis.

The normality of the distribution was verified by the Shapiro–Wilk test. The distribution of the analyzed quantitative variables was not normal. The non-parametric Kruskal–Wallis test was used to assess the statistical significance of more than two groups of independent variables without a normal distribution. The variability of the parameters was presented in the form of the median and the interquartile range (IQR). Spearman’s correlation test was used to assess the correlation between the examined parameters, taking into account the level of statistical significance. The existing correlations are presented using a correlation matrix. The results at the level of *p* < 0.05 were considered statistically significant. In order to analyze many factors per variable in the studied groups, multiple linear regression was used. The variability in the parameters was presented as the regression coefficient and the standard error of the regression coefficient, using a statistical significance level of *p* < 0.005.

## 3. Results

### 3.1. Characteristics of the Studied Population

The women in the study were 31 (4) years of age with a BMI of 2.81 (5.18). The WHR of women was 0.81 (0.08), and 48.64% (*n* = 107) of the participants were primiparous. In total, 73.18% (n = 161) had a vaginal delivery, and 68.18% (n = 150) lived in cities in Kujawsko-Pomorskie Voivodeship.

The characteristic of the study groups is presented in Table 1.

The age and WHR of breastfeeding women did not differ statistically significantly between the study groups. A higher BMI value was shown in the group of women breastfeeding from 2 to 8 weeks compared to breastfeeding over 49 weeks of lactation (*p* = 0.025) (Table 2).

### 3.2. The Basic Composition of Human Milk

**Figure 2:**
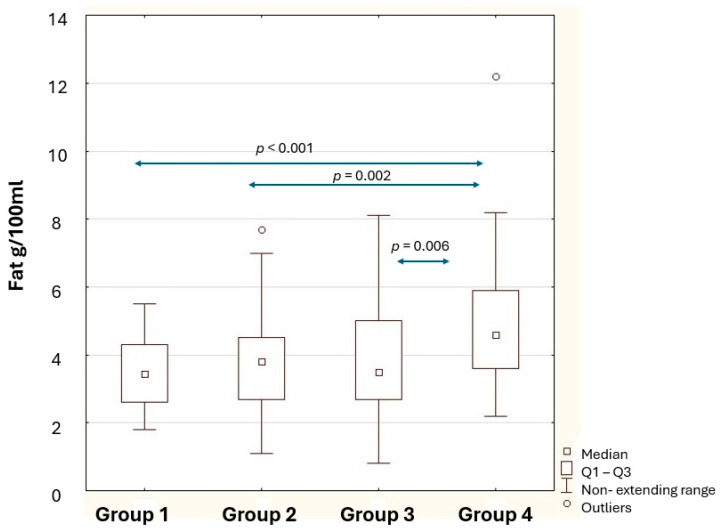
Statistical significance of differences in human milk fat content between the study groups. Group 1—breastfeeding 2–7 weeks; Group 2—breastfeeding 8–24 weeks; Group 3—breastfeeding 25–48 weeks; Group 4—breastfeeding 49–96 weeks; *p*—level of statistical significance; Q1–Q3—first quartile and third quartile.

The basic composition of Polish women’s milk changes significantly with the lactation period (Table 3). The fat content of human milk varied with lactation time. The highest concentration was in Group 4, while the lowest was in Group 1 (Figure 2). In the initial lactation period, the level of fat in human milk was 25% lower compared to the longest lactation period (*p* < 0.001). The fat content of milk was significantly different between samples from breastfeeding women from 6 to 12 months (Group 3) compared to milk from women breastfeeding from 1 year to 2 years (Group 4) (*p* = 0.006).

**Figure 3:**
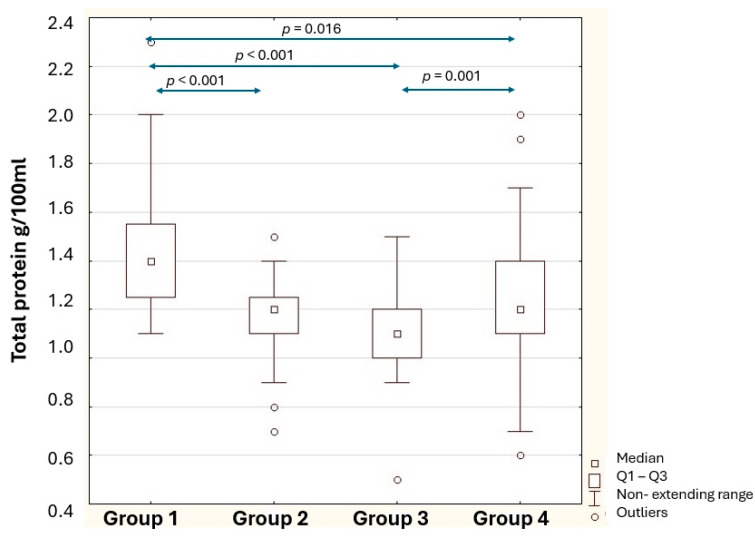
Statistical significance of differences in total protein of human milk content between the study groups. Group 1—breastfeeding 2–7 weeks; Group 2—breastfeeding 8–24 weeks; Group 3—breastfeeding 25–48 weeks; Group 4—breastfeeding 49–96 weeks; *p*—level of statistical significance; Q1–Q3—first quartile and third quartile.

The concentration of total protein in human milk was highest in Group 1 (Figure 3). Milk from 8 weeks to 6 months of age (Group 2) showed a 16.67% lower total protein content compared to the previous lactation period (*p* < 0.001). A lower protein content of total milk in women breastfeeding from six months to a year (Group 3) by 27.27% compared to Group 1 (*p* < 0.001) was shown. The total human milk protein levels in Group 4 increased by 9.09% compared to Group 3 (*p* = 0.001). In addition, a decrease in carbohydrates in Group 4 by about 4% was observed compared to Group 3 (*p* = 0.007).

### 3.3. Hormones in Human Milk

No statistically significant differences in the concentration of adiponectin or cortisol were found in the tested human milk samples (Table 4). Lower leptin concentrations were observed in Group 4 and Group 3 compared to Group 1 (*p* = 0.001/ *p* = 0.015).

### 3.4. Antioxidant Status of Human Milk

**Figure 4:**
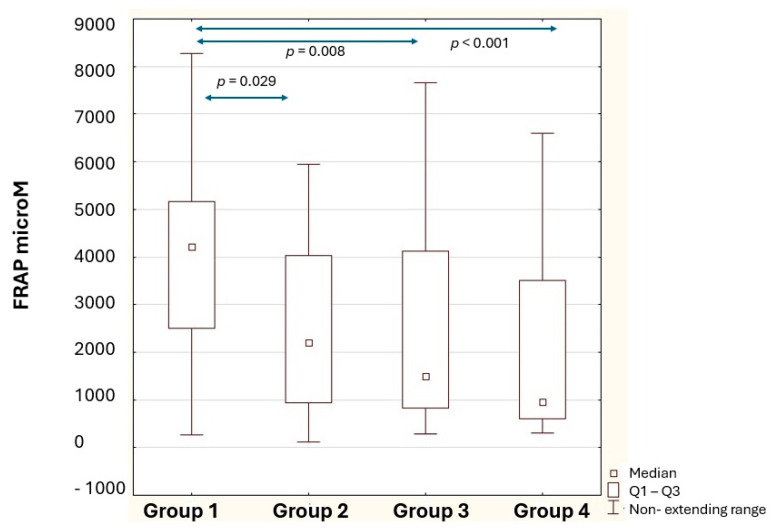
Statistical significance of differences in human milk FRAP levels in the study groups. Group 1—breastfeeding 2–7 weeks; Group 2—breastfeeding 8–24 weeks; Group 3—breastfeeding 25–48 weeks; Group 4—breastfeeding 49–96 weeks; *p*—the level of satirical significance; Q1–Q3—first quartile and third quartile.

The antioxidant status varies significantly depending on the stage of lactation (Table 5). A decreased ability of human milk to reduce iron ions with lactation was observed. The highest level of FRAP was shown at the beginning of lactation (Group 1); it was higher by 47.86% compared to Group 2, by 180.60% compared to Group 3, and by 344.66% compared to Group 4 (Figure 4). In addition, a 17.83% higher level of milk DPPH was observed in Group 1 compared to Group 4 (*p* = 0.021). No significant differences were observed in the antioxidant status of the milk of women breastfeeding from six months to a year (Group 3) compared to milk from women breastfeeding up to 2 years (Group 4).

### 3.5. Vitamins and Micro- and Macroelements in Human Milk

No significant statistical differences in the concentration of chlorides, phosphorus, or calcium in human milk were observed in the studied groups (Table 6). Lower iron concentrations in human milk were shown in Group 2 (*p* = 0.018) and Group 4 (*p* = 0.001) compared to Group 1. In the milk of women breastfeeding up to 2 years of age (Group 4), a higher concentration of the magnesium by 33.61% was observed compared to the beginning of lactation (*p* = 0.001).

Numerous correlations between the investigated parameters in the studied groups were demonstrated, which are presented in Table 7, Table 8, Table 9 and Table 10.

### 3.6. Multiple Linear Regression

Due to the possible influence of factors other than lactation on the composition of human milk, multiple linear regression was performed. Table 11 shows the factors that showed a statistically significant impact on the variability of parameters in the studied groups.

## 4. Discussion

In this paper, we focused on the variability of human milk composition and antioxidant status in samples from women living in Poland. Our results complement the existing literature describing changes in milk composition depending on the duration of lactation. Additionally, our findings provide further data on the antioxidant status of human milk from women breastfeeding for more than 12 months. According to scientific research, the basic composition of human milk changes throughout lactation [34,35,36]. Our results also confirm this relationship.

We observed a higher BMI in women who were breastfeeding from 2 to 7 weeks compared to women who were breastfeeding for more than 1 year. No differences were found in parameters such as HBD or WHR. Our results are similar to those observed by Mandel et al. (2005) and Lubetzky et al. (2012) [36,37]. The higher BMI in breastfeeding mothers in the early stages of lactation compared to control groups is due to the state of pregnancy, as these women are in the postpartum period and their bodies are returning to their pre-pregnancy state. Additionally, studies suggest that breastfeeding promotes weight loss and reduces WHR [38].

The presence of older mothers in the BF > 12 group may indicate significant socioeconomic differences between the two groups [36].

### 4.1. Variability in the Basic Composition of Milk

We showed higher levels of fat in human milk from women breastfeeding >12 months compared to human milk from women breastfeeding from 6 to 12 months (*p* = 0.006), women breastfeeding from 8 weeks to 6 months (*p* = 0.004), and women breastfeeding from 2 to 7 weeks (*p* < 0.001). Similar results were presented by Ongprasert et al. (2020) [34], Lubetzky et al. (2012) [37], Mandel et al. (2005) [36], and Czosnykowska-Łukacka et al. (2018) [35]. Muts et al. (2025)suggest. that the fat content in human milk increases from the eighth month of lactation [39]. Lubetzky et al. (2012) additionally examined the variability of fatty acid composition during lactation. They showed that the percentage of total fatty acids (except C12 and C14) decreased significantly with the duration of lactation, in contrast to C12:0 and C14:0, which increased [37]. Czosnykowska-Łukacka et al. showed that the fat content significantly increased in HM expressed by mothers lactating beyond 18 months postpartum, whereas Shehadem et al. [38] and Perrin et al. [40] concluded that fat concentration was not related to lactation duration.

We observed the highest concentration of total protein in human milk in the initial lactation period, then this parameter gradually decreased. On the other hand, in the milk of women who were breastfeeding for more than 12 months, the concentration of total protein increased compared to the milk of women who breastfed from 6 to 12 months (*p* = 0.001). Our results are supported by the research of Muts et al. (2025). The authors showed that the concentration of total protein in human milk follows a U-shaped path throughout lactation [39]. Interestingly, Czosnykowska-Łukacka et al. (2018) showed that total protein concentration increased in the second year after delivery and was higher than in pooled milk samples from donors breastfeeding for less than a year. The authors of the study suggest that the increase in total protein levels in human milk may be due to the increase in immunoglobulin and lactoferrin concentrations in milk during lactation [23,35].

Additionally, we observed lower levels of carbohydrates in human milk in the BF > 12 group compared to the 6–12 BF group. This is confirmed by Czosnykowska-Łukacka et al. (2018) [35]. Muts et al. (2025) suggest that the carbohydrate content of human milk is stable throughout lactation [39].

Some changes in concentration may be related to the amount of milk produced, so fat concentrations may increase as lactation progresses. The reason for this may be smaller volume and less full breasts. Lower levels of carbohydrates (including lactose) and higher levels of protein and fat may indicate a reduction in the volume of milk produced [41].

### 4.2. Variability in Human Milk Antioxidant Status

Our studies showed a decline in the antioxidant status of human milk, including the ability of human milk to reduce iron ions over lactation time. Similar conclusions were described by Quiles et al. 2006; Alberti-Fidanza et al. 2002; and Buescher & McIlheran 1988 [42,43,44]. Zarban et al. (2009) showed that the average total antioxidant capacity of colostrum using the FRAP method is 1061.6 ± 500.6 µM, transitional milk 915.3 ± 511.4 µM, and mature milk 724.7 ± 302.4 µM. In addition, the researchers assessed the ability of human milk to reduce DPPH• radicals, showing the following average results: for colostrum 50.4 ± 19.7%, for transitional milk 40.8 ± 20.0%, and for mature milk 38.2 ± 17.3% [45]. Despite the declining antioxidant activity of human milk, protection against free radicals is still maintained at a good level.

These data suggest that the use of colostrum in the first days of life is crucial due to its high antioxidant potential. Furthermore, research by Mehta et al. demonstrated that bioactive proteins enhance the antioxidant potential of human milk, which is lacking in commercial formulas, and this study confirms that human milk is an ideal food for infants [44,46].

The results of our own research on the antioxidant status of human milk with the use of DPPH, FRAP, and polyphenol concentration showed that there are no differences between the milk of women breastfeeding >12 months and the milk of women breastfeeding from 6 to 12 months. These results are confirmed by Ongprasert et al. (2020), who assessed the total antioxidant capacity of human milk. Based on limited research, the highest levels of antioxidant components and TAC (total antioxidant capacity) can be concluded to be present in colostrum, which then decrease during early lactation [34].

### 4.3. Variability of Selected Hormones and Micro- and Macronutrients of Human Milk

Our study included analyses of adiponectin, leptin, and cortisol concentrations in human milk in relation to the lactation period. As of today, this is a pioneering study, as there is no literature describing the variability of the above-mentioned hormones during lactation over a year. We showed a decrease in leptin content in human milk in women breastfeeding for more than 12 months (*p* = 0.001) and in women breastfeeding from 6 to 12 months (*p* = 0.015) compared to human milk from early lactation.

Leptin concentrations in breast milk vary throughout lactation and show significant interindividual differences among breastfeeding mothers [47,48]. Maternal characteristics appear to be key factors influencing this variability, with factors such as body composition, nutritional status, hormonal balance, and environmental and physiological conditions being associated with the modulation of leptin levels in breast milk [49]. In our study, we observed positive correlations between maternal BMI and breast milk leptin concentrations. Leptin levels in breast milk typically reflect maternal leptin levels and metabolic status, potentially acting as a regulator of infant appetite. However, evidence regarding leptin concentrations in breast milk from mothers giving birth preterm compared with those giving birth at term remains conflicting in the literature. While some studies report higher leptin levels in breast milk from mothers giving birth preterm, others report lower levels or no significant differences compared to breast milk from mothers giving birth at term. Ilcol et al. (2006) confirmed the variability of leptin concentration with lactation duration. They found that milk leptin concentration was highest in colostrum and decreased during the first 180 days of lactation [50]. These inconsistent results reveal a clear gap in knowledge regarding the effect of gestational age at delivery on leptin concentration in the milk of full-term mothers.

Our study did not show statistically significant differences in the concentrations of adiponectin and cortisol in the analyzed samples. Gridneva et al. (2018) examined adiponectin and leptin levels in the milk of women breastfeeding for up to 12 months and similarly reported no statistically significant differences in these parameters [51].

Our study also gains particular value through the inclusion of an analysis of selected micro- and macroelements in human milk during prolonged lactation. We demonstrated that the highest iron concentration in milk occurs in women breastfeeding at the beginning of lactation. Later, the level stabilizes, and there are no statistically significant differences between the milk of women breastfeeding for 6–12 months and that of women breastfeeding for more than 12 months. Raj et al. (2008), who analyzed iron and lactoferrin concentrations in human milk, reported a decrease in iron levels as lactation progressed [52].

Shashiraj et al. showed that the iron content in human milk is highest in early transitional milk (0.97 mg/mL) but steadily decreases during lactation, reaching a level of approximately 0.35 mg/mL in the first month of lactation to 0.20 mg/mL in the sixth month [53]. In the study by Raj et al., from birth to six months, the mean iron concentration in human milk ranged from 0.89 to 0.26 mg/L in mothers without anemia (group A) and from 0.86 to 0.33 mg/L in mothers with anemia (group B). The mean lactoferrin concentration in the milk of mothers without anemia (group A) was 12.02 mg/mL, 5.84 mg/mL, and 5.85 mg/mL at 1 day, 6 weeks, and 6 months, respectively, while in mothers with anemia (group B) at the same time points it was 12.91 mg/mL, 6.68 mg/mL, and 6.37 mg/mL, respectively. Importantly, no significant differences were found in the concentration of iron or lactoferrin in the milk of mothers without anemia and mothers with anemia at 1 day, 14 weeks, and 6 months after delivery [49].

In our study, we observed a higher concentration of magnesium in the milk of women who were breastfeeding for over 12 months compared to early lactation periods. However, we did not observe changes in phosphorus, chloride, or calcium concentrations in milk with lactation. Feeley et al. (1983) did not observe any changes when studying these elements [54].

### 4.4. Strengths and Limitations of the Study

Despite the increasing number of studies on the nutrients and composition of human milk, studies assessing antioxidant status, selected hormones, and micro/macronutrients according to the stage of lactation are still lacking. This highlights the novelty of our study. Research beyond 6 months postpartum is needed, based on evidence that human milk composition changes over the 24-month period, and that extended breastfeeding provides additional benefits for infant growth and development. Our results are significant because they indicate that human milk 12 months postpartum contains increased concentrations of fat and total protein and is characterized by a stable antioxidant status.

However, we acknowledge several limitations. We did not assess milk volume or 24 h milk intake by infants; therefore, the precise amounts consumed by infants could not be determined. This, combined with the large variability in lactation duration after 12 months, limits our ability to fully interpret the effects of these nutrients on infant outcomes. Another limitation of our study was the relatively small sample size of 220 patients. No statistical calculations of group size or test power were used in selecting the sample sizes. Our study employed convenience sampling due to the inclusion and exclusion criteria for study participants.

It should be emphasized, however, that our study group was divided into four groups with a comparable number of participants. The final group consisted of long-term breastfeeding women (49–96 weeks of breastfeeding). Currently, in Poland, a small percentage of women breastfeed for more than a year, which is why the groups have limitations in terms of size.

Olczak-Kowalczyk et al. demonstrated that long-term breastfeeding mothers in Poland are one of the most criticized social groups. The study results indicate that as many as 63% of breastfeeding women encountered unfavorable comments and were discouraged from breastfeeding. In total, 76% were criticized, insulted, or ridiculed by family or friends, and 75% received unpleasant comments or recommendations from healthcare professionals, 28% of which were from pediatricians [55].

Being aware of the limitations of our work, in the future we would like to expand the study to include a larger number of participants (including women from all over Poland), as well as to analyze the composition and antioxidant status of human milk from different periods of lactation, taking into account the volume of milk.

## 5. Conclusions

Human milk composition after 12 months postpartum is characterized by higher fat and total protein content, along with maintained antioxidant protection. Although the concentrations of many bioactive and nutritive components continue to increase as lactation progresses, the daily intake of some of these components may remain stable despite the reduction in overall milk consumption by the infant. Human milk beyond 12 months continues to provide protection and support developmental programming during the challenging period of solid food introduction and rapid infant growth. Therefore, continued breastfeeding past 12 months and beyond is encouraged.

## Figures and Tables

**Table 1:** The characteristics of the study groups.

	Group 1 *N* = 40	Group 2 *N* = 52	Group 3 *N* = 65	Group 4 *N* = 63
Age (years)	30 (5.5)	30 (4)	30 (3)	32 (3)
BMI (kg/m^2^)	25.18 (3.84)	23.42 (3.9)	22.26 (4.98)	21.80 (4.97)
WHR	0.82 (0.09)	0.81 (0.07)	0.81 (0.07)	0.79 (0.09)
HBD	40 (1)	39.5 (1)	40 (1)	40 (1)
Fertility	*N* (%)			
Primiparous	*N* = 15 (37.5%)	*N* = 23 (44.23%)	*N* = 34 (52.31%)	*N* = 35 (55.55%)
Multiparous	*N* = 25 (62.5%)	*N* = 29 (55.77%)	*N* = 31 (47.69%)	*N* = 28 (44.45%)
Delivery	*N* (%)			
Vaginal	*N* = 31 (77.5%)	*N* = 42 (82.77%)	*N* = 40 (61.54%)	*N* = 48 (76.19%)
Cesarean Section	*N* = 9 (22.5%)	*N* = 10 (17.23%)	*N* = 25 (38.46%)	*N* = 19 (23.81%)
Residence	*N* (%)			
City	*N* = 27 (67.5%)	*N* = 32 (61.54%)	*N* = 48 (73.85%)	*N* = 43 (68.25%)
Village	*N* = 13 (32.5%)	*N* = 20 (38.46%)	*N* = 17 (26.15%)	*N* = 21 (31.75%)

Values for age, BMI, WHR, and HBD shown as median and IQR.

**Table 2:** Statistical significance for the characteristics of the studied groups.

	*p* (1/2)	*p* (2/3)	*p* (3/4)	*p* (1/3)	*p* (1/4)	*p* (2/4)
Age	0.999	0.999	0.590	0.999	0.265	0.385
BMI	0.999	0.999	0.999	0.074	0.025	0.442
WHR	0.999	0.999	0.730	0.999	0.483	0.821

Kruskal–Wallis test; *p*—level of statistical significance between groups; 1,2,3,4—study group. Group 1—breastfeeding 2–7 weeks; Group 2—breastfeeding 8–24 weeks; Group 3—breastfeeding 25–48 weeks; Group 4—breastfeeding 49–96 weeks; red color indicates statistical significance.

**Table 3:** Basic composition of human milk depending on the duration of lactation.

	Group 1 *N* = 40	Group 2 *N* = 52	Group 3 *N* = 65	Group 4 *N* = 63	*p* 1/2	*p* 2/3	*p* 3/4	*p* 1/3	*p* 1/4	*p* 2/4
Fat g/100 mL	3.45 (1.7)	3.8 (1.8)	3.5 (2.3)	4.6 (2.3)	0.999	0.999	0.006	0.999	<0.001	0.002
Total protein g/100 mL	1.4 (0.3)	1.2 (0.15)	1.1 (0.2)	1.2 (0.3)	<0.001	0.335	0.001	<0.001	0.016	0.186
Carbohydrates g/100 g	7.5 (0.85)	7.5 (0.75)	7.9 (0.5)	7.6 (0.63)	0.986	0.234	0.007	0.006	0.999	0.999
Total solids g/100 g	12.6 (2.35)	12.95 (2.4)	12.7 (2.5)	13.4 (2.9)	0.999	0.999	0.999	0.999	0.390	0.267
Energy value kcal/100 g	69.5 (18.15)	71 (21)	68 (21)	75 (27)	0.999	0.999	0.181	0.999	0.175	0.159

Kruskal–Wallis test; values shown using the median (IQR); *p*—level of statistical significance; Red color indicates that statistical significance was demonstrated.

**Table 4:** Concentration of selected hormones in human milk samples depending on the duration of lactation.

	Group 1 *N* = 40	Group 2 *N* = 52	Group 3 *N* = 65	Group 4 *N* = 63	*p* 1/2	*p* 2/3	*p* 3/4	*p* 1/3	*p* 1/4	*p* 2/4
Adiponectin pg/mL	3499 (2775.5)	3211 (2842)	3189 (2910)	3850 (3557)	0.999	0.999	0.999	0.999	0.999	0.999
Leptin pg/mL	31.41 (32.48)	18 (33.83)	15.6 (30.67)	2.32 (30.63)	0.233	0.999	0.999	0.015	0.001	0.406
Cortisol ng/mL	8.22 (3.45)	7.27 (4.21)	7.68 (6.13)	8.56 (4.4)	0.999	0.999	0.999	0.999	0.999	0.462

Kruskal–Wallis test; values shown by the median (IQR); *p*—level of statistical significance; Red color indicates that statistical significance was demonstrated.

**Table 5:** Parameters of the antioxidant status of human milk depending on the duration of lactation.

	Group 1	Group 2	Group 3	Group 4	*p* 1/2	*p* 2/3	*p* 3/4	*p* 1/3	*p* 1/4	*p* 2/4
DPPH % inhibition	71.31 (13.11)	65.68 (28.61)	67.76 (33.71)	60.52 (24.09)	0.240	0.999	0.621	0.739	0.021	0.999
FRAP µM	4214.4 (2658.6)	2197.12 (3080.96)	1501.89 (3290.66)	947.77 (2888.46)	0.029	0.999	0.599	0.008	0.001	0.382
Polyphenols mg GAE/L	16.89 (141.02)	9.63 (15.01)	10.33 (12.35)	10.98 (11.37)	0.036	0.999	0.999	0.069	0.366	0.999

Kruskal–Wallis test; values shown by median (IQR); *p*—level of statistical significance; FRAP—iron ion reduction ability; DPPH—2,2-diphenyl-1-picrylhydrazyl radical reduction method; GAE—gallic acid.

**Table 6:** Concentration of selected vitamins. micro- and macroelements in human milk samples depending on the duration of lactation.

	Group 1 *N* = 40	Group 2 *N* = 52	Group 3 *N* = 65	Group 4 *N* = 65	*p* 1/2	*p* 2/3	*p* 3/4	*p* 1/3	*p* 1/4	*p* 2/4
Iron µg/dl	67.20 (49.35)	38.34 (40.93)	39.71 (43.81)	37.29 (46.37)	0.018	0.999	0.345	0.063	0.001	0.999
Magnesium mg/dl	2.36 (0.2)	2.53 (1.67)	2.55 (1.5)	3.18 (1.95)	0.239	0.999	0.464	0.100	0.001	0.313
Phosphorus mg/dl	7.26 (2.21)	6.54 (2.17)	6.77 (2.41)	6.06 (2.77)	0.150	0.999	0.999	0.702	0.053	0.999
Chlorides mmol/l	9.79 (6.01)	9.53 (5.78)	9.78 (6.42)	11.26 (6.34)	0.999	0.999	0.935	0.999	0.999	0.498
Calcium mg/dl	10.28 (2.13)	11.07 (4.69)	10.85 (4.42)	10.65 (6.53)	0.999	0.999	0.999	0.999	0.999	0.769

Kruskal-Wallis test; values shown by median (IQR); *p* – level of statistical significance.

**Table 7:** Spearman rank order correlation matrices significant with *p* < 0.05 in Group 4 (*n* = 63).

	BMI	Leptin	Cortisol	DPPH	FRAP	Fat	Total Protein	Carbohydrates	Total Solid	Energy Value	WHR	Polyphenols	Iron	Magnesium	Phosphorus	Chlorides	Calcium
Age	0.033	0.197	0.112	0.093	0.029	−0.002	0.032	−0.015	0.095	0.101	−0.004	0.049	0.071	−0.014	0.163	0.140	−0.036
BMI	1.000	0.241	−0.118	−0.108	0.184	0.317	0.036	0.110	0.135	0.131	0.435	0.029	0.040	0.125	0.035	−0.176	0.038
Adiponectin	0.051	0.062	−0.111	0.073	0.064	0.117	0.025	−0.094	0.075	0.072	−0.015	−0.140	−0.041	0.093	0.109	−0.015	0.006
Leptin	0.241	1.000	0.242	0.463	0.626	−0.113	−0.088	−0.180	−0.035	−0.017	0.348	0.117	0.610	−0.619	0.594	−0.340	−0.363
Cortisol	−0.118	0.242	1.000	0.061	0.029	−0.118	−0.315	0.123	−0.043	−0.048	0.054	−0.120	0.154	−0.260	−0.007	−0.138	−0.024
DPPH	−0.108	0.463	0.061	1.000	0.428	0.033	0.224	−0.065	0.143	0.153	0.062	0.062	0.283	−0.351	0.396	−0.189	−0.447
FRAP	0.184	0.626	0.029	0.428	1.000	−0.428	−0.064	−0.194	−0.341	−0.334	0.182	0.126	0.456	−0.540	0.541	−0.306	−0.425
Fat	0.317	−0.113	−0.118	0.033	−0.428	1.000	0.289	0.155	0.818	0.834	0.144	−0.080	−0.299	0.435	−0.209	0.074	0.155
Total protein	0.036	−0.088	−0.315	0.224	−0.064	0.289	1.000	−0.078	0.424	0.380	−0.059	0.111	−0.123	0.207	−0.031	0.161	−0.100
Carbohydrates	0.110	−0.180	0.123	−0.065	−0.194	0.155	−0.078	1.000	0.280	0.127	0.103	−0.184	−0.109	0.124	−0.137	−0.073	0.205
Total solids	0.135	−0.035	−0.043	0.143	−0.341	0.818	0.424	0.280	1.000	0.975	0.072	−0.043	−0.161	0.286	−0.053	0.080	0.074
Energy value	0.131	−0.017	−0.048	0.153	−0.334	0.834	0.380	0.127	0.975	1.000	0.015	−0.034	−0.169	0.258	−0.010	0.059	0.027
WHR	0.435	0.348	0.054	0.062	0.182	0.144	−0.059	0.103	0.072	0.015	1.000	0.289	0.148	0.034	−0.126	0.085	0.028
HBD	−0.043	−0.016	0.380	−0.014	−0.183	−0.041	0.044	0.002	−0.036	−0.030	−0.048	0.112	−0.006	−0.089	−0.040	0.170	−0.127
Polyphenols	0.029	0.117	−0.120	0.062	0.126	−0.080	0.111	−0.184	−0.043	−0.034	0.289	1.000	0.196	−0.002	0.115	0.278	−0.115
Iron	0.040	0.610	0.154	0.283	0.456	−0.299	−0.123	−0.109	−0.161	−0.169	0.148	0.196	1.000	−0.600	0.415	−0.277	−0.154
Magnesium	0.125	−0.619	−0.260	−0.351	−0.540	0.435	0.207	0.124	0.286	0.258	0.034	−0.002	−0.600	1.000	−0.566	0.404	0.531

The “−” sign next to the value indicates an inverse correlation; the lack of a sign indicates a direct correlation; Red text color represents statistical significance.

**Table 8:** Spearman rank order correlation matrices significant with *p* < 0.05 in Group 3 (*n* = 65).

	Leptin	Cortisol	DPPH	FRAP	Fat	Total Protein	Carbohydrates	Total Solids	Energy Value	WHR	Iron	Magnesium	Phosphorus	Chlorides	Calcium
Age	−0.060	−0.093	−0.123	−0.114	−0.187	0.019	−0.151	−0.225	−0.247	0.202	0.212	0.080	−0.174	0.001	−0.016
BMI	−0.040	−0.050	0.187	−0.034	0.172	−0.005	0.045	0.126	0.080	0.120	0.103	−0.015	−0.060	−0.204	0.203
Adiponectin	0.046	0.140	−0.102	0.008	−0.003	0.130	−0.135	−0.010	−0.061	−0.079	−0.097	0.076	0.012	0.087	0.097
Leptin	1.000	0.186	0.636	0.677	0.142	0.158	0.159	0.250	0.268	0.032	0.488	−0.777	0.328	−0.224	−0.666
Cortisol	0.186	1.000	0.124	0.276	0.211	0.265	0.063	0.227	0.217	0.088	−0.051	−0.085	0.357	−0.114	−0.075
DPPH	0.636	0.124	1.000	0.601	0.378	0.303	0.183	0.446	0.422	−0.141	0.409	−0.603	0.428	−0.186	−0.439
FRAP	0.677	0.276	0.601	1.000	0.208	0.409	0.059	0.240	0.314	0.010	0.545	−0.743	0.461	−0.220	−0.633
Fat	0.142	0.211	0.378	0.208	1.000	0.176	0.183	0.929	0.840	−0.327	0.069	−0.194	0.160	−0.161	−0.191
Total protein	0.158	0.265	0.303	0.409	0.176	1.000	0.061	0.293	0.136	0.011	0.218	−0.149	0.169	−0.077	−0.224
Carbohydrates	0.159	0.063	0.183	0.059	0.183	0.061	1.000	0.387	0.357	0.044	0.016	−0.075	0.053	−0.272	−0.161
Total solids	0.250	0.227	0.446	0.240	0.929	0.293	0.387	1.000	0.845	−0.315	0.119	−0.259	0.226	−0.223	−0.253
Energy value	0.268	0.217	0.422	0.314	0.840	0.136	0.357	0.845	1.000	−0.234	0.124	−0.284	0.184	−0.156	−0.288
WHR	0.032	0.088	−0.141	0.010	−0.327	0.011	0.044	−0.315	−0.234	1.000	0.405	−0.048	−0.292	−0.075	0.105
Polyphenols	−0.028	−0.043	−0.153	−0.024	−0.241	0.178	−0.079	−0.179	−0.142	0.094	0.097	0.084	−0.153	0.023	0.066
Iron	0.488	−0.051	0.409	0.545	0.069	0.218	0.016	0.119	0.124	0.405	1.000	−0.649	0.145	−0.244	−0.347
Magnesium	−0.777	−0.085	−0.603	−0.743	−0.194	−0.149	−0.075	−0.259	−0.284	−0.048	−0.649	1.000	−0.380	0.434	0.736
Phosphorus	0.328	0.357	0.428	0.461	0.160	0.169	0.053	0.226	0.184	−0.292	0.145	−0.380	1.000	−0.112	−0.293
Chlorides	−0.224	−0.114	−0.186	−0.220	−0.161	−0.077	−0.272	−0.223	−0.156	−0.075	−0.244	0.434	−0.112	1.000	0.364

The “−” sign next to the value indicates an inverse correlation; the lack of a sign indicates a direct correlation; Red text color represents statistical significance.

**Table 9:** Spearman rank order correlation matrices significant with *p* < 0.05 in Group 2 (*n* = 52).

	Age	BMI	Leptin	Cortisol	DPPH	FRAP	Fat	Total Protein	Carbohydrates	Total Solids	Energy Value	WHR	Polyphenols	Iron	Magnesium	Phosphorus	Chlorides	Calcium
Age	1.000	0.008	0.283	0.034	0.186	0.250	0.128	−0.287	0.044	0.166	0.144	0.187	−0.009	0.060	−0.358	0.178	−0.145	−0.138
BMI	0.008	1.000	−0.045	−0.155	−0.014	−0.051	0.167	−0.121	−0.053	0.100	0.129	0.347	0.054	−0.027	−0.109	−0.161	−0.014	0.237
Adiponectin	−0.025	−0.193	0.202	0.003	−0.079	0.005	−0.179	−0.105	−0.088	−0.170	−0.147	−0.182	0.146	0.133	−0.119	0.256	−0.054	0.054
Leptin	0.283	−0.045	1.000	0.303	0.659	0.685	0.042	−0.164	−0.298	−0.063	0.090	0.149	0.075	0.518	−0.767	0.423	−0.414	−0.578
Cortisol	0.034	−0.155	0.303	1.000	0.010	−0.015	−0.140	0.048	−0.279	−0.168	−0.114	0.044	−0.100	0.128	−0.210	0.248	−0.181	−0.095
DPPH	0.186	−0.014	0.659	0.010	1.000	0.577	0.097	−0.174	−0.118	0.018	0.137	0.143	−0.100	0.279	−0.646	0.306	−0.425	−0.576
FRAP	0.250	−0.051	0.685	−0.015	0.577	1.000	0.096	−0.043	−0.257	−0.003	0.082	0.025	0.005	0.423	−0.603	0.325	−0.370	−0.638
Fat	0.128	0.167	0.042	−0.140	0.097	0.096	1.000	−0.006	0.237	0.931	0.941	0.045	−0.006	0.037	−0.145	0.165	−0.042	0.001
Total protein	−0.287	−0.121	−0.164	0.048	−0.174	−0.043	−0.006	1.000	0.164	0.109	0.057	−0.128	−0.187	−0.244	0.383	−0.054	0.260	0.019
Carbohydrates	0.044	−0.053	−0.298	−0.279	−0.118	−0.257	0.237	0.164	1.000	0.492	0.328	0.126	−0.272	−0.188	0.139	−0.169	−0.129	0.149
Total solids	0.166	0.100	−0.063	−0.168	0.018	−0.003	0.931	0.109	0.492	1.000	0.934	0.011	−0.015	−0.059	−0.040	0.096	0.011	0.042
Energy value	0.144	0.129	0.090	−0.114	0.137	0.082	0.941	0.057	0.328	0.934	1.000	0.074	−0.015	0.001	−0.153	0.160	−0.050	−0.045
WHR	0.187	0.347	0.149	0.044	0.143	0.025	0.045	−0.128	0.126	0.011	0.074	1.000	−0.256	0.149	−0.188	−0.012	−0.262	−0.024
Polyphenols	−0.009	0.054	0.075	−0.100	−0.100	0.005	−0.006	−0.187	−0.272	−0.015	−0.015	−0.256	1.000	0.306	0.052	−0.162	0.376	0.027
Iron	0.060	−0.027	0.518	0.128	0.279	0.423	0.037	−0.244	−0.188	−0.059	0.001	0.149	0.306	1.000	−0.582	0.315	−0.263	−0.239
Magnesium	−0.358	−0.109	−0.767	−0.210	−0.646	−0.603	−0.145	0.383	0.139	−0.040	−0.153	−0.188	0.052	−0.582	1.000	−0.394	0.655	0.547
Phosphorus	0.178	−0.161	0.423	0.248	0.306	0.325	0.165	−0.054	−0.169	0.096	0.160	−0.012	−0.162	0.315	−0.394	1.000	−0.183	−0.164
Chloride	−0.145	−0.014	−0.414	−0.181	−0.425	−0.370	−0.042	0.260	−0.129	0.011	−0.050	−0.262	0.376	−0.263	0.655	−0.183	1.000	0.481

The “−” sign next to the value indicates an inverse correlation; the lack of a sign indicates a direct correlation; Red text color represents statistical significance.

**Table 10:** Spearman rank order correlation matrices significant with *p* < 0.05 in Group 1 (*n* = 40).

	Age	Adiponectin	Leptin	Cortisol	DPPH	FRAP	Fat	Total Protein	Carbohydrates	Total Solids	Energy Value	WHR	Iron	Magnesium	Calcium
Age	1.000	0.072	0.388	−0.004	−0.099	0.092	−0.126	−0.005	0.125	−0.063	−0.027	0.365	0.171	−0.147	−0.253
BMI	0.061	−0.023	0.050	0.260	0.325	0.251	−0.071	0.088	−0.088	−0.100	−0.049	0.054	0.100	−0.171	0.082
Adiponectin	0.072	1.000	0.241	−0.065	0.075	0.180	−0.202	0.090	0.156	−0.050	−0.006	0.272	0.067	−0.414	−0.337
Leptin	0.388	0.241	1.000	−0.333	0.175	0.314	−0.203	0.031	−0.138	−0.159	−0.048	0.300	0.307	−0.471	−0.462
Cortisol	−0.004	−0.065	−0.333	1.000	0.027	0.223	−0.171	−0.122	−0.250	−0.331	−0.274	0.167	0.162	0.091	−0.094
DPPH	−0.099	0.075	0.175	0.027	1.000	0.426	−0.250	−0.037	−0.361	−0.352	−0.290	0.108	0.215	−0.339	−0.362
FRAP	0.092	0.180	0.314	0.223	0.426	1.000	−0.609	−0.388	−0.347	−0.641	−0.549	0.160	0.339	−0.434	−0.504
Fat	−0.126	−0.202	−0.203	−0.171	−0.250	−0.609	1.000	0.307	0.238	0.872	0.825	0.150	−0.195	0.296	0.371
Total protein	−0.005	0.090	0.031	−0.122	−0.037	−0.388	0.307	1.000	0.247	0.523	0.514	0.270	−0.238	0.052	0.307
Carbohydrates	0.125	0.156	−0.138	−0.250	−0.361	−0.347	0.238	0.247	1.000	0.611	0.411	−0.122	−0.321	0.166	0.158
Total solids	−0.063	−0.050	−0.159	−0.331	−0.352	−0.641	0.872	0.523	0.611	1.000	0.903	0.102	−0.336	0.275	0.370
Energy value	−0.027	−0.006	−0.048	−0.274	−0.290	−0.549	0.825	0.514	0.411	0.903	1.000	0.203	−0.278	0.185	0.316
WHR	0.365	0.272	0.300	0.167	0.108	0.160	0.150	0.270	−0.122	0.102	0.203	1.000	0.381	−0.064	−0.120
Polyphenols	−0.222	0.076	0.098	−0.057	0.284	−0.078	0.194	0.191	−0.183	0.107	0.079	−0.032	0.359	−0.118	−0.073
Iron	0.171	0.067	0.307	0.162	0.215	0.339	−0.195	−0.238	−0.321	−0.336	−0.278	0.381	1.000	−0.303	−0.301
Magnesium	−0.147	−0.414	−0.471	0.091	−0.339	−0.434	0.296	0.052	0.166	0.275	0.185	−0.064	−0.303	1.000	0.404
Phosphorus	−0.200	−0.041	0.213	−0.013	0.055	0.163	0.147	−0.023	−0.124	0.069	0.215	0.041	0.183	−0.197	0.128
Chlorides	−0.239	−0.180	−0.061	−0.047	0.060	−0.189	0.329	0.201	0.063	0.244	0.139	−0.012	0.080	0.374	0.316
Calcium	−0.253	−0.337	−0.462	−0.094	−0.362	−0.504	0.371	0.307	0.158	0.370	0.316	−0.120	−0.301	0.404	1.000

The “−” sign next to the value indicates an inverse correlation; the lack of a sign indicates a direct correlation; Red text color represents statistical significance.

**Table 11:** Results of multiple linear regression between the examined parameters (cortisol, phosphorus, magnesium, leptin, fat, energy value, polyphenols, and DPPH) and predictors (residence, BMI, age, and WHR) in the study groups.

Group 1 *N* = 40		Cortisol	Phosphorus	DPPH	Group 2 *N* = 52	Leptin	Magnesium	Polyphenols
Residence	B	0.486	0.568			0.496	−0.331	
	SE	0.226	0.211			0.132	0.141	
	P	0.041	0.012			0.001	0.025	
BMI	B		−0.538					
	SE		0.203					
	P		0.013					
Age	B			0.397		0.281		
	SE			0.188		0.133		
	P			0.045		0.041		
WHR	B							−0.455
	SE							0.150
	P							0.004
Group 3 *N* = 65		DPPH	Fat	Energy value	Phosphorus	Group 4 *N* = 63	Fat	
Residence	B	0.283	0.317	0.292				
	SE	0.130	0.121	0.126				
	P	0.035	0.011	0.024				
WHR	B		−0.392	−0.291	−0.289			
	SE		0.128	0.133	0.143			
	P		0.012	0.034	0.049			
BMI	B		0.321				0.462	
	SE		0.123				0.150	
	P		0.003				0.003	

B—regression coefficient; SE—standard error from the regression coefficient; *p*—statistical significance level < 0.050.

## Data Availability

Data will be made available upon request. Data are stored at the Department of Pathobiochemistry and Clinical Chemistry. Collegium Medicum in Bydgoszcz (Poland). Person responsible for providing data: Agnieszka Chrustek (mail: a.chrustek@cm.umk.pl).

## Abbreviations

BF	breastfeeding
BMI	body mass index
DHA	docosahexaenoic-acid
DPPH	2,2-diphenyl-1-picrylhydrazyl radical
EPA	eicosapentaenoic acid
FRAP	iron ion reduction capacity
GAE	gallic acid
HBD	hebdomas
*p*	level of statistical significance
Q_1_–Q_3_	first and third quartile
WHR	waist–hip ratio
